# Protecting infrastructure performance from disinformation attacks

**DOI:** 10.1038/s41598-022-16832-w

**Published:** 2022-07-26

**Authors:** Saeed Jamalzadeh, Kash Barker, Andrés D. González, Sridhar Radhakrishnan

**Affiliations:** 1 School of Industrial and Systems Engineering, University of Oklahoma, Norman, OK 73019 USA; 2 School of Computer Science, University of Oklahoma, Norman, OK 73019 USA

**Keywords:** Civil engineering, Energy grids and networks

## Abstract

Disinformation campaigns are prevalent, affecting vaccination coverage, creating uncertainty in election results, and causing supply chain disruptions, among others. Unfortunately, the problems of misinformation and disinformation are exacerbated due to the wide availability of online platforms and social networks. Naturally, these emerging disinformation networks could lead users to engage with critical infrastructure systems in harmful ways, leading to broader adverse impacts. One such example involves the spread of false pricing information, which causes drastic and sudden changes in user commodity consumption behavior, leading to shortages. Given this, it is critical to address the following related questions: (i) How can we monitor the evolution of disinformation dissemination and its projected impacts on commodity consumption? (ii) What effects do the mitigation efforts of human intermediaries have on the performance of the infrastructure network subject to disinformation campaigns? (iii) How can we manage infrastructure network operations and counter disinformation in concert to avoid shortages and satisfy user demands? To answer these questions, we develop a hybrid approach that integrates an epidemiological model of disinformation spread (based on a susceptible-infectious-recovered model, or SIR) with an efficient mixed-integer programming optimization model for infrastructure network performance. The goal of the optimization model is to determine the best protection and response actions against disinformation to minimize the general shortage of commodities at different nodes over time. The proposed model is illustrated with a case study involving a subset of the western US interconnection grid located in Los Angeles County in California.

## Introduction

Well-publicized disinformation campaigns surrounding recent US Presidential elections and the adoption of pandemic-related vaccinations have increased awareness among researchers that historical problems of misinformation / disinformation are exacerbated due to the wide availability and use of online platforms. Disinformation, defined as information that falsely characterizes the state of the system, including rumors, factual errors, and attempts at deception^1^, is rising on online platforms^2,3^.

There is substantial literature on modeling the effects of and protection against false data injections by adversaries and connections to the operability and functionality of critical infrastructures^4–6^. However, an over-the-horizon problem may result from an adversary that seeks to attack critical infrastructure indirectly by altering the consumption behavior of human intermediaries who are influenced by weaponized disinformation distributed by the adversary.

Consider the following plausible scenarios that are extended from collections of actual events. An airline passenger could tweet an alert about a suspicious package, which, if shared rapidly and widely, could cause significant delays in flights and major traffic jams on many primary, secondary, and tertiary roads (similar to what was experienced at London’s Gatwick airport^7^). Hackers could compromise a major US pipeline network, but the rampant spread of misinformation leads to a dramatic escalation in the aftermath and a physical, real-world increase in gas prices (similar to what was experienced when a news network spread a false story about a Russian hack of the US power grid^8^). Finally, false reports of accidents on social media could lead to dynamic rerouting of drivers, causing congestion in particular areas subject to attack (similar events have occurred globally^9–11^ and could worsen with the emergence of autonomous vehicles^12^).

To begin to address some of these scenarios, we develop a model to examine the interactions between information/disinformation spread, subsequent commodity consumption behavior, and the resulting infrastructure network balance. To do so, we integrate an epidemiological model of information/disinformation spread with a network flow model. We relate the two models through human intermediaries who adopt information/disinformation that changes the way that they interact with the infrastructure network.

Contributions of our integrated model include: (i) we account for the evolution of disinformation spread over time based on the outcomes of virtual interaction between pair of users in social media, ultimately projecting user behavior onto commodity consumption, (ii) we introduce an information protection mechanism to combat against disinformation spread, and (iii) we develop a mixed-integer programming formulation to balance the performance of the critical infrastructure network and plan for targeting (good) information to counter disinformation.

This paper is organized as follows. The Background and Literature section provides a methodological background on the concepts of disinformation spread and critical infrastructure network optimization along with the associated literature. The subsequent model section explains the proposed integration of epidemiological and mathematical programming models. The Case Study section illustrates the proposed model with a case study involving the power distribution network in Los Angeles County, California. Finally, the Conclusion section offers concluding remarks and future research opportunities.

## Background and literature review

Our proposed work relies on two key areas: (i) spread of information and disinformation, and (ii) network flow models for infrastructure. In this section, we offer a review of these two areas, detailing some of the current research gaps addressed in this paper.

### Models of information and disinformation spread

Within social networks, people exchange information to ultimately influence others, where influence is defined as an action “to induce a change in the behavior of another that is in accordance with the wishes of the influencer”^13,14^. Each individual communicates with (or influences) many other peers and, similarly, individuals are influenced by numerous other peers. This influence can take on negative forms, such as information pollution, fake news, propaganda, misinformation, misinformation, disinformation, and hoaxes^15–17^. Social media users can become a source of online broadcast activity that affects personal and social behavior. Users may help speed up the transfer rate of information or disinformation and manipulate the content to match their points of view, deliberately or inadvertently, which may not be necessarily verified or verifiable. In such an online environment, if users do not pay enough attention to verified content and reliable sources of information, the information they receive may have varying levels of correctness and malicious intent.

The community of users can be classified into different categories based on how they respond to the influence of others. An analogy to the spread of influence and different categories of response is the spread of disease and different states of infection found in epidemiology literature^18^. A basic model for the spread of disease is the susceptible-infected-recovered (SIR) model, which uses a series of differential equations to describe the membership of different states at a point in time: those who are susceptible to the disease, those who are infected by it, and those who have recovered from it. An analogy can be made for those users reacting to information and disinformation. For example, for a group of power utility users who receive a fake message promoting a discount price for power usage during a specific time, those users may potentially share it with others or not, based on characteristics (e.g., personal traits) they exhibit. Using the SIR convention, individuals who adopt this disinformation and react to it directly by consuming more power can be classified as “Infected.” Alternatively, users who are not influenced by this disinformation, for any reason, can be classified as “Removed.” And users who have not received notification yet can be classified as “Susceptible.” This classification of categories allows us to model, quantify, and predict their power usage during disinformation dissemination. There is a rich literature that formulates the phenomenon of transition between categorical labels with SIR models that employ a system of differential equations based on mean field theory or agent-based models that allow us to simulate the transmission of disinformation among autonomous agents in a flexible microscale manner^19–21^.

Social media users are not limited to categories S, I, and R. For example, some groups of users intend to spread accurate information to fight against disinformation, or those who have already received disinformation but do not reshare it with other users, or those who have received disinformation but temporarily do not share it^22–24^. There may be communities on social media that spread authenticated information to counteract disinformation^22,25^. Furthermore, users in each category (that is, S, I, and R) can be classified as *aware* and *unaware*, where it is assumed that unaware users can become aware users based on contact with aware individuals at a given rate. Still, it is assumed that the reverse transition will not occur. We define the terminology “awareness” as knowledge and understanding that something is happening or exists^26^. In addition to the novel categories attached to classic SIR models to represent a community, some methods are developed to avoid bias originating from discretizing the solutions of SIR models^27^.

Several different derivatives of the SIR class of models have been developed to extend the various categories of adoption of influence (e.g., information and disinformation)^28^. Given the link between networks and the spread of diseases^29,30^, the SIR modeling enterprise has applications in other network-related applications: the spread of ideas^31–34^ and the influence of social networks^35–39^. A related idea by^40^ uses a variation of the SIR model to address the stifling of rumors. Still, it does not adequately allow for the competitive nature to describe the spread of information versus disinformation. It is because disinformation spreads differently than information, as noted in^41^, with the former spreading faster and covering a large population on Twitter^2^. Social responses to disinformation will be examined by observing (i) how people evaluate information, (ii) how varying situations affect people’s ability to evaluate information effectively, and (iii) how people act on information, including redistributing disinformation. In our context, *S* refers to individuals who have not yet been exposed to the disinformation content, *I* represents individuals who have heard the disinformation and changed their consumption behavior as a result, and *R* represents individuals who have heard the disinformation but ignored them after realizing that the information they received was not true or accurate.

### Models of infrastructure flow optimization

Complex infrastructure systems such as water, gas, transportation, and electricity are crucial for society’s well-being and for promoting economic productivity. If one component of the system is affected by failure, larger spread effects can be experienced in other networks of infrastructures and networks of community members that suffer from unmet demand for goods and services^42^. As such, the resilience of critical infrastructure networks attracted researchers to study the ability of systems to mitigate the magnitude and duration of the components of the out-of-service infrastructure network^43,44^.

Flow balance models are developed to determine how commodities are delivered from suppliers to customers, so that performance metrics such as average unsupplied demand and transportation costs are minimized, while guaranteeing that key operational constraints are observed. By the term “commodity,” we broadly refer to flows of demanded entities (e.g., electric power, water, vehicles, data, goods) transmitted from one node to another through links connecting them. Several different flow balance optimization models are proposed in the literature that are applicable to infrastructures focused on disruptive events^45^.

In the literature, there are numerous representations of network optimization of infrastructure networks. Hsu et al.^46^ presented a generalized network flow model to model the long-term supply and demand of water resources. Tahiri et al.^47^ proposed a network flow optimization model for similar water distribution networks, minimizing the total cost of meeting the demand for water. Martin et al.^48^ optimized a gas network consisting of a set of compressors and pipes that connect the valves in order to minimize the total cost of the network subject to supply-demand balance. Banda et al.^49^ similarly proposed a gas pipeline network optimization model that accounted for nonlinear isothermal equations. Traffic flow optimization problems have also been proposed^50^. Darayi et al.^51^ proposed a multicommodity network flow optimization model to understand the criticality of different multimodal transportation nodes and links.

Especially important to the case study addressed subsequently are network optimization problems designed for electric power networks. Vasin et al.^52^ proposed a model to optimize the flow of energy resources through a transportation network. Costa et al.^53^ developed a two-stage linear programming model to reinforce power grids against attacks on transmission lines, proposing an exact algorithm to solve the model. Leuthold et al.^54^ developed a nonlinear mixed-integer programming model to design an electricity market such that public welfare is maximized, with an application to the European electricity market. Wirtz et al.^55^ proposed a sustainable multicommodity system design model with the power grid attached to the system using mixed integer linear programming. Electric power networks are critical sources of energy that enable the function of other infrastructures, and developing flow balance optimization models for electric power grids has become important for researchers^56^, as have several network flow optimization representations of interdependent infrastructure networks that include electric power^57–60^.

In this paper, we address the challenge of how to track and respond to disinformation attacks that disrupt infrastructure networks. Embedding the evolution of disinformation diffusion intensity over time attached to a flow balance optimization model of infrastructure network has two main benefits: (i) we can monitor and analyze the performance of infrastructure network disrupted by disinformation attacks over time, and (ii) act in opposition to disinformation propagation to mitigate the effect of disruption on the infrastructure network performance. To the best of our knowledge, such a model has not been proposed in the literature yet. To address this gap, we propose a network flow balance optimization model integrated with disinformation diffusion model that enables us to take opposite actions using social media to handle interruptions in infrastructure networks caused by disinformation attack.

## Proposed integrated epidemiological + optimization (EPO) model

We propose and integrate two models to examine the interactive relationship between disinformation dissemination and critical infrastructure network performance: (i) the SIR model and (ii) a network flow balance optimization model. The network balance optimization model is used to balance critical infrastructure systems with respect to disinformation propagating on social networks, as the spread of disinformation on social networks affects the consumption behavior of social network users. These two networks are integrated in a multi-to-one environment from the social network to the critical infrastructure network, where communities of users are assigned to the set of infrastructure nodes.

### SIR model

We describe the disinformation propagation process in the type of modeling “compartmental models” in which the population of social media users is divided into exclusive compartments. In such a formulation, we assign the rates at which the population within one compartment is transferred to another. In general, we can classify users into three exclusive compartments over time: (i) *S*, the proportion of users who are unaware of the disinformation and would have acted on it if known, (ii) *I*, the proportion of users who consumed the disinformation and changed (acted on the disinformation) their commodity usage schedule, and (iii) *R*, the proportion of users who were exposed to the disinformation but either ignored or detected it and are not interested in sharing it. Dividing the population of each compartment, we can formulate the dynamics of the compartments by replacing the size of the population with the proportion of the population.

The rate of transfer from one state to another is expressed as derivatives of the proportion of population in terms of time, and we make some assumptions to express the terms of the model. As such, we have a system of differential equations that describe how the proportion of people changes across different states over time by frequent communication. For example, given a population size *N*, for an unaware user randomly communicating with other users, the probability that the unaware user meets a user who adopted disinformation can be expressed by $\frac{I}{N}$, and the rate of contact can be described as a coefficient of the total population, $\beta N$. Assuming that meetings between unaware users, *S*, and users who adopted disinformation result in the unaware user adopting disinformation, the population of unaware users decreases by $\beta S I$, and the population of users who adopted disinformation increases by the same size in time slots. Through this transformation process, users who consume disinformation have the opportunity to detect or ignore disinformation at a rate $\gamma$, with the associated population $\gamma I$, and are no longer classified as the group that already consumes disinformation. Thus, the population size that adds up to ignorant users is expressed as $\gamma I$ that are removed from users who consume and adopt disinformation.

We can formulate the SIR compartmental models by introducing more compartments such as hesitants, who can be the users who have not yet decided to adopt the disinformation or not. Although the introduction of new compartments can simulate the real-world information transfer process more accurately, tweaking the parameters of the basic SIR model results in different transformation evolution paths at a relatively lower computational cost. For this reason, the basic SIR model is sufficient to generate different evolutions of users who adopted disinformation at a reasonable computational cost. Estimating the proportion of population who adopted disinformation results in estimating the evolution of commodity demand changes over time horizon of interest in our proposed model.

The evolution of the proportion of these groups over time is modeled on the basis of the homogeneous SIR model, which is mathematically represented by the system of differential equations (a.k.a. mean-field equations) (1)–(3) subject to constraint (4).

1 $$\begin{aligned}&\frac{dS_{i,t}}{dt} = -\beta S_{i,t} I_{i,t}, \; \forall i \in V, \; \forall t \in T, \end{aligned}$$

2 $$\begin{aligned}&\frac{dI_{i,t}}{dt} = \beta S_{i,t} I_{i,t} - \gamma I_{i,t}, \; \forall i \in V, \; \forall t \in T, \end{aligned}$$

3 $$\begin{aligned}&\frac{dR_{i,t}}{dt} = \gamma I_{i,t}, \; \forall i \in V, \; \forall t \in T, \end{aligned}$$

4 $$\begin{aligned}&S_{i,t} + I_{i,t} + R_{i,t} = 1, \; \forall i \in V, \; \forall t \in T. \end{aligned}$$

The index $i \in V$ represents the community surrounding node *i*, and $t \in T$ denotes time. Under the assumption of homogeneity, users are equally likely to interact with other users. Also, we assumed that no users leave their interactions (e.g., leave social media) during the time of analysis. Therefore, the sum of proportions of the three categories remains constant and equals 1.

The user status can change from one state to another over time. A susceptible (unaware) user encounters an infected (disinformed) user that is infected at a rate $\beta$, and a user can move from state *I* to *R* by detecting disinformation at a rate $\gamma$. That is, in essence, $\beta$ governs the rate at which disinformation spreads, and $\gamma$ governs the rate at which disinformed users recover their behavior. Our approach is motivated by interactions on social networks^61^. However, since the parameters of the model, $\gamma$ and $\beta$, govern the rate at which disinformation spreads, other means of social interaction (e.g., TV, radio, web forums) can be taken into account with appropriate rate parameter settings.

There are several ways to solve our system of equations such as the Euler and Runge-Kutta (RK) methods and their derivatives^62^. Each method has advantages and disadvantages in terms of accuracy order and computational cost. The SIR model that we have deployed in our analysis is a non-linear model that needs to be solved numerically by multi-stage algorithms to return the estimates with reasonable accuracy. The forward Euler method is a special case of the RK method, so it solves our problem with relatively low accuracy. At the expense of computational cost, we found the RK algorithm suitable for solving our nonlinear system in terms of accuracy^63,64^.

### Network flow balance optimization model

**Table 1:** Model notation.

Notation	Description
**Sets**	
*V*	Set of infrastructure network nodes
*E*	Set of infrastructure network links
*T*	Set of periods
**Parameters**	
$\bar{t}_t$	Duration of each period starting from time *t* to the beginning of its next period
$q_{it}$	The amount of supply in node $i \in V$ at time $t \in T$
$m_{ijt}$	Capacity of link from node *i* to node *j* at time $t \in T$
$p_{it}$	Community size surrounding the node *i* at time $t \in T$
$d^c_{it}$	Commodity consumption per capita by the community surrounding the node *i* at time $t \in T$
$r^p_{it}$	Proportion of commodity consumption of the community surrounding the node $i \in V$ at time $t \in T$ responsive to price shift
$\rho _{it}$	Estimated sensitivity of commodity consumption of the community surrounding the node $i \in V$ at time $t \in T$ based on the price shift
$\dot{I}_{it}$	Local derivative (change per unit of time interval $\bar{t}_t$) of the proportion of community surrounding node $i \in V$ targeted by disinformation at time $t \in T$
$r_{it}$	The proportion at which $\dot{I}_{i,t}$ can be changed by spreading counter (good) information for the community surrounding the node $i \in V$ at time $t \in T$
$n^p_t$	Total number of target locations informed by counter (good) information at time $t \in T$
**Decision variables**	
$x_{ijt}$	The amount of transmitted commodity (flow) from node $i \in V$ to node $j \in V$ at time $t \in T$
$h_{it}$	Shortage (undersupplied) amount of commodity at node $i \in V$ at time $t \in T$
$e_{it}$	Excess (oversupplied) amount of commodity at node $i \in V$ at time $t \in T$
$d_{it}$	Nominal demand of commodity in node $i \in V$ at time $t \in T$
$I_{it}$	Proportion of community surrounding node $i \in V$ at time $t \in T$ adopted disinformation
$g_{it}$	=1 if counter (good) information is released for the surrounding community in node $i \in V$ at time $t \in T$; =0 otherwise

Mathematical programming has proven to be an efficient approach to model and optimize engineered systems and processes^65,66^. Network flow balance optimization can be formulated into a mathematical programming model. There are different ways to formulate network flow optimization problems, however, some formulations are more efficient to solve in terms of complexity^67^. Mathematical programming problems are classified based on the type of decision variables, constraints, and objective functions used in the model. To reduce the computation costs of highly complex problems, there exist some reformulations, which help optimization algorithms to iterate relatively faster or converge to optimal solutions with relatively lower iterations. Among these models, linear programming models are polynomially solvable, while integer and mixed-integer programming models (e.g., the models with integer decision variables) are mostly computationally more expensive to solve^68^. Thus, modeling a problem in linear format is much better in terms of computational complexity. If integer variables need to be included in the model, there are reformulation techniques to convert or divide the models to smaller problems to be solved faster. We formulated the network flow balance optimization problem efficiently and as simple as possible to include a relatively low number of integer decision variables. As a result, we could solve the model iteratively in a reasonable amount of time to compare the results of the optimization problem with respect to different values of model parameters.

We model the infrastructure network as a graph *G*(*V*, *E*), where the set of nodes, *V*, represents the nodes incorporating demand, supply, and transmission nodes. The set of links, *E*, represents the links that connect the nodes. There is a link between the nodes if there is a transmission line to transmit the commodity. With the notation found in Table 1, the following is a mixed integer programming (MIP) model to protect the performance of the critical infrastructure network against disinformation dissemination.

5 $$\begin{aligned}&\underset{x,h,e,d,I,g}{\mathrm {min}} \sum _{i \in V, \; t \in T}{ h_{it} } \end{aligned}$$

6 $$\begin{aligned}&\text {s.t.} \nonumber \\&\sum _{j \in V : (j,i)\in E} x_{jit} - \sum _{k \in V : (i,k)\in E} x_{ikt} + h_{it} - e_{it} + q_{it} - d_{it} = 0, \qquad \forall i \in V, \forall t \in T, \end{aligned}$$

7 $$\begin{aligned}&x_{ijt} \le m_{ijt}, \qquad \forall i \in V, \forall j \in V, \forall t \in T, \end{aligned}$$

8 $$\begin{aligned}&d_{it} = p_{it} d^c_{it} \big \{ ( 1-I_{it} ) + \{ I_{it} [ r^p_{it} (1+\rho _{it}) + (1 - r^p_{it}) ] \} \big \}, \qquad \forall i \in V, \forall t \in T, \end{aligned}$$

9 $$\begin{aligned}&I_{i, t+\bar{t}_t} = I_{i t} + \dot{I}_{i,t+\bar{t}_t} ( 1 - r_{it} \; g_{it} ), \qquad \forall i \in V \setminus \{ \mid V \mid \}, \forall t \in T, \end{aligned}$$

10 $$\begin{aligned}&\sum _{i \in V} g_{it} \le n^p_t, \qquad \forall t \in T, \end{aligned}$$

11 $$\begin{aligned}&x_{ijt}, h_{it}, e_{it} \in \mathbb {R}_{\ge 0}, \qquad d_{it}, I_{it} \in \mathbb {R}_{\ge 0}, \qquad g_{it} \in \{ 0,1 \}. \end{aligned}$$

Equation (5) is the objective function that minimizes the total amount of commodity shortage resulting from altered consumption behavior over time. Constraint (6) guarantees the balance of the input, output, produced and consumed of the commodity for all nodes. The balance equations are implicitly borrowed from the model proposed by Tang et al.^69^. Constraint (7) limits the capacity of the links. Constraint (8) represents the baseline and responsive demand in terms of the number of users targeted for disinformation given the elasticity of the commodity demand with respect to exogenous factors (e.g., discount price message). Constraint (9) is used to account for the counter- spread of good information as a strategy to control disinformation dissemination. Constraint (10) limits the number of nodes to focus information countering strategies. The last set of constraints (11) describes the nature of the decision variables.

Solutions to this optimization problem can guide decisions to mitigate an commodity shortage based on disinformation, namely: (i) the amount of commodity flow that should be transmitted through the links, (ii) the optimal shortage or excess in each node, and (iii) the optimal number and location of our communities (surrounding particular nodes) to spread counter information to prevent the adverse effects of disinformation campaigns.

Note that a node cannot experience a shortage and excess simultaneously at the node level. We assume that social media users react to disinformation logically. For example, once a false price discount disinformation is broadcast, social network users consume more commodity relative to their baseline usage.

## Case study: performance of the electric power network under disinformation attack

An electric power system is a network of electrical nodes, such as power plants, transformers, or demand points, connected by links that represent transmission lines, cables, or transformers. In such networks, the nodes represent the equipment of the power system and the links are the pathways for the transmission of electrical energies. The electrical energies that are transmitted are called power flows. Electrical power networks are used to satisfy the needs of load nodes (demand nodes) anywhere in the network by transferring the electric power produced by generators (supply nodes) through the links in the network. Each link can carry the maximum commodity through the network, called the flow capacity. Therefore, flow transmissions are limited due to flow capacities in the network. Since the flow capacities are finite in power systems, the problem of transferring the flows to satisfy the demand nodes is a vital network optimization problem that needs to be studied.

To balance the electric power system, several different models and methods are proposed in the literature, such as mathematical optimization and machine learning^70^. For example, Nasrolahpour et al.^71^ developed a mixed-integer programming model to alleviate electric power congestion in transmission lines to ultimately minimize the electric power shortage and total cost. Clack et al.^72^ developed a linear programming model for electric power balance given its engineering requirements. In the models mentioned above, the common constraint of concern for the authors was a system-wide constraint to guarantee the balance between supply and demand nodes over the network. Also, to obtain realistic solutions to the model, the capacity of the transmission line is specified before optimizing the model. We utilize similar constraints from the literature and include a mechanism to counter disinformation dissemination to defend the spread of disinformation.

In recent years, a handful of papers have begun to address the potential for disinformation to affect commodity consumption. Nguyen et al.^73^ developed a vulnerability assessment model to mitigate the adverse effects of disinformation on load shedding. Tang et al.^69^ developed an optimization model to minimize total load shedding in a power network under the condition that users react to price disinformation, relating those reactions to user personality traits. Raman et al.^6^ developed an attacker-defender optimization model to mitigate strategic urban power distribution system attacks based on price disinformation (e.g., falsely offering prizes for rescheduled power usage) propagated through the community based on the “Believe, Accept, and Follow Through” mechanism.

Among all critical infrastructures, the electric power grid has been attractive to scholars for several reasons: (1) the electric power grid has been at great risk of attack and threats tremendously^74^; (2) electric power grid has been relatively more expensive than other infrastructures^75^; (3) the electric grid is one of the most vital infrastructure during disasters (e.g., Hurricane Sandy in New York) since it is an indirect critical source of commodity for other vital sectors^56^. For these reasons, the analysis of the performance of power grids under dissemination of disinformation has attracted the most attention.

Although we offer a general modeling approach that can be manipulated for a variety of critical infrastructures, our case study is motivated by the electric power grid. Disruptions to power distribution systems can result in substantial economic and social costs^76^. Due to various social factors (e.g., human mistakes, irrationality, intentional gaming, malicious attacks), the electric power grid may become more vulnerable to various kinds of cyber and physical activities when social information becomes tightly integrated into its operation. For example, a coordinated attack could cause a significant impact, such as that experienced in the Ukrainian power grid in 2015^77^.

Electric power utilities are increasingly taking advantage of *demand response* programs to reduce or shift electricity usage during peak periods in response to time-based rates or other forms of financial incentives to customers^69,78^. Such demand response programs will be important in the future grid^79,80^. Demand response messaging has been primarily textual, coming from text messages, emails, or other social media messages^6,81^. Naturally, these messages affect human consumption behavior and are used to run an efficient electrical power grid system. Unfortunately, this valuable and effective mechanism could also be used to spread *disinformation*, thus creating a weapon to create a harmful effect - disrupting the power system. Imagine a Tweet being spread by a realistic but fake Twitter account. A discount price is offered to those whose power usage exceeds their average daily use by 30% during summer afternoons. As more and more customers (even those who are not the creators of the disinformation) spread this disinformation and subsequently adopt its message, blackouts will occur more likely due to overloads in the system, along with broader spread impacts to public health and safety. If a threshold of users in a particular geographical location adopts disinformation, a disruption in the power network will occur.

### Determining the parameters of the model

The proportion of social media users who may adopt disinformation is found in the SIR model as the proportion of users that make up the group *S*.

To evaluate the relationship between this spread of disinformation and the demand for electricity, we must also estimate the change in the demand for electricity driven by users whose consumption changed based on disinformation. The elasticity of the use of electric power measures the responsiveness of the electric power demanded to a change in price. Data from the US state level show that the estimated residential price elasticity of electric power demand is −0.7, suggesting that residential electricity consumption is inelastic to price changes^82,83^. A well-known formula, the midpoint method, used to compute the elasticity of electric power demand is found in Eq. (12), where $pr_{i,t}$ represents the price of the electric power utility at node $i\in V$ at time $t \in T$. As a result, the parameter $\rho _{i,t}$ estimates the proportional change in electric power usage around node $i \in V$ at time $t \in T$ based on a false discount price message.

12 $$\begin{aligned} \rho _{i,t} = \frac{ d_{i,t+1} - d_{i,t} }{ pr_{i,t+1} - pr_{i,t} } \times \frac{pr_{i,t+1} + pr_{i,t}}{d_{i,t+1} + d_{i,t}} \end{aligned}$$

Disinformation messages can take different forms to affect electricity use, such as fake weather conditions, fake availability, false prices of alternative sources of electricity (e.g., coal, oil, renewable sources), and false announcements that describe the general economic situation. Such factors have been shown to significantly influence electric power consumption^84,85^ with different effects depending on geography and spatiotemporal aggregation^82,83^.

The management of loads in electric power systems is highly dependent on the retail price and sales of electricity. Broadcasting false price discount signals on social networks results in increased electricity use by a large number of consumers at once, which may eventually lead to overload or blackout at the node or system level. However, when users who choose to adopt the false pricing message receive it, not all electrical power usage changes accordingly. That is, not all electric power usage is responsive to price changes. For example, price changes might affect a more variable usage appliance (e.g., air conditioner usage may increase) relative to one that is not as variable (e.g., a refrigerator will use the same amount of electricity regardless). Based on the US Energy Information Administration, we assume that 92% of electricity use is responsive to price changes and the rest is consumed to meet basic needs of life^86^. Therefore, we assume that a false discount price message can only affect 92% of electricity usage.

Additionally, not everyone has the ability to receive disinformation messages on social networks. The reports reveal that 82% of the US population were social media users in 2021^87^. Therefore, we assumed that in each population surrounding electric power buses, only 82% people have access to social media directly and are susceptible to receiving the disinformation message. Among these communities, not all residents are sensitive to disinformation about the change in electric price, as not all family members are responsible for household decisions. It is shown that the composition of the home is an influential factor in determining electricity consumption^88^. Thus, we adjust the community $p_{it}$ accordingly to incorporate the effective proportion of the population who may be more responsible for household decisions and who may be more at risk of adopting disinformation.

In summary, we assumed that a fake discount price message can affect 82% of users, and among the total electricity usage they consume, only 92% of their usage may change based on the disinformation they receive (if they adopt the disinformation). As a result, the demand per capita, $d^c_{i,t}$, is modified in the model accordingly.

Designers of electric power transmission systems should ensure that the system can operate normally under unanticipated loads. The safety factor is used to tolerate the system to meet unexpected loads and avoid waste of energy resources. Furthermore, the safety factor of electric power transmission systems is a measure of transmission line reliability that accounts for the possibility of overloaded electric power flow through transmission lines. Due to the lack of data, we use a primary Linear Programming (LP) model with no specified capacity to estimate the capacity of the transmission lines. Assuming that the power distribution lines operate optimally, we take 0.2 ($\pm 20 \%$) as a proportion of the allowed volatility of the power flow in the transmission lines. We set the estimated values as the capacity of the transmission lines.

The model we proposed to estimate the capacity of transmission lines is as the following LP problem in Eqs. (13)–(16), where $\bar{d}_{i,t}$ is the nominal demand of the population assigned to the bus *i* at time *t*.

13 $$\begin{aligned}&\underset{x,h,e}{\mathrm {min.}} \sum _{i \in V, \; t \in T}{ h_{it} } \end{aligned}$$

14 $$\begin{aligned}&\text {s.t.} \end{aligned}$$

15 $$\begin{aligned}&\sum _{j \in V : (j,i)\in E} x_{jit} - \sum _{k \in V : (i,k)\in E} x_{ikt} + h_{it} - e_{it} + q_{it} - \bar{d}_{it} = 0, \qquad \forall i \in V, \forall t \in T \end{aligned}$$

16 $$\begin{aligned}&x_{ijt}, h_{it}, e_{it} \in \mathbb {R}_{\ge 0} . \end{aligned}$$

### Numerical results

We applied the proposed model to evaluate the effect of disinformation on electric power distribution systems. The electric power nodes supply the electricity to its surrounding community. We overlay the geospatial population data with the topology of the power network to establish the boundary of the model. We use the US Census Application Programming Interface (API) to collect geospatial population data surrounding each electrical power node^89^. The American Community Survey (ACS) provides population data, for use in the model. In addition, we spatially clipped power nodes and links (that is, we generated a shapefile based on spreadsheet data provided by the synthetic power grid data set^90^) that intersected with Los Angeles (LA) County block groups and overlaid it with LA County population data. Approximately 1600 people live in each block group in LA County.

**Figure 1:**
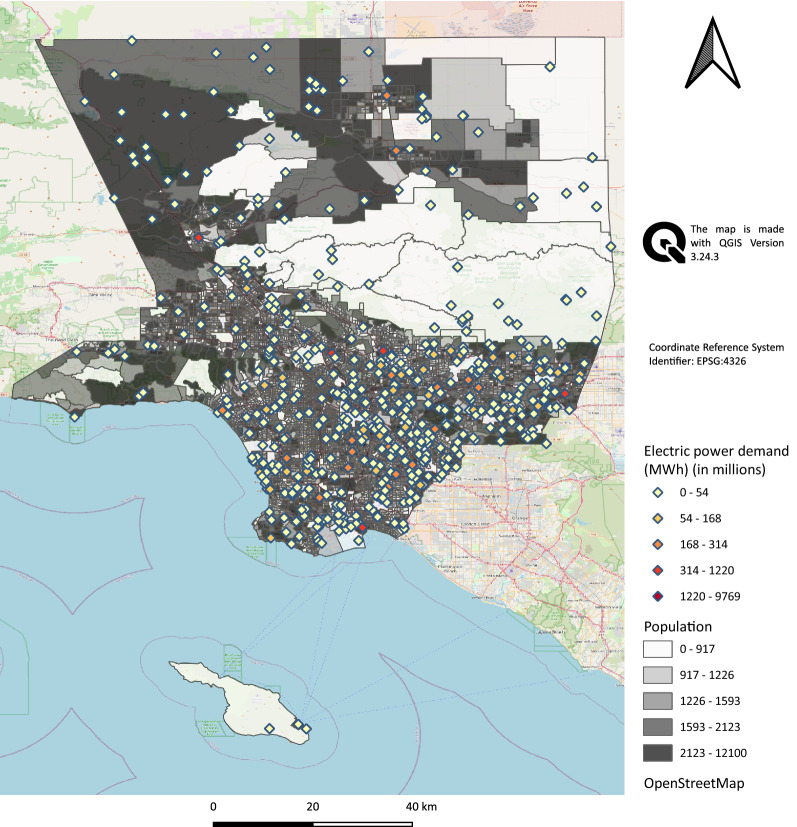
Distribution of electric power demand and population, LA County, USA.

For homeland security purposes, the actual topology of the power grid is not publicly available. However, the information on topology, demand and supply is estimated using *network imitating method based on learning* (NIMBLE)^91^, resulting in a publicly accessible synthetic power grid data set for the western interconnection grid of the United States^90^. We limited the boundaries of this larger power distribution system to LA County in California. It incorporates more than 500 power buses (nodes) and 600 transmission lines (links), the geospatial dispersion of which is shown in Fig. 1.

Although studying the effect of disinformation evolution on critical infrastructures at the micro-level makes more sense, highly detailed information is not publicly available. For example, while studying the evolution of disinformation integrated with low-voltage electric power systems may be more desired, we may be more able to estimate parameters of high-voltage electric power systems across a relatively larger geographical area (e.g., the treatment of the impacts of disinformation on a high-voltage network by^92^).

A portion of commodity consumers are assumed to be users of social networks. We relate consumer products to spatially defined block groups, defined as a statistical division of US Census tracts that consists of clusters of blocks that generally contain 600 to 3000 residents of the contiguous area^93^, and each group of consumers is linked to the nodes explained.

To relate social media users to electric power buses, we performed geospatial operations for these two features. First, we defined two sets that incorporate electric power buses and aggregated social media users. Electric power buses and social networks are geographically represented by points and polygonal features, respectively. Social media users live in census block groups, defined as a statistical division of US Census tracts that consists of clusters of blocks that generally contain 600 to 3000 residents of the contiguous area^93^. Then we calculated the Euclidean distance matrix between the electrical power buses and the centroid of polygons. As a result, block groups are assigned to one power bus according to their shortest Euclidean distance, and several block groups are mapped to electric power buses and are characterized by estimates of power usage. As such, SIR models are deployed for each social media user within each block group.

Based on the estimated usage of social networks in 2021^87^, it is assumed that 82% of the population in each block group have active access to social networks, so they are potentially susceptible to being targeted by disinformation. To run the disinformation propagation model, we considered 1% population being targeted by disinformation at the beginning of the analysis time period. As time goes on, the proportion of susceptible, infected (targeted) and recovered users changes according to the contact rate, the rate of being targeted by disinformation, and the rate of being aware of disinformation.

The topology of the power distribution network and the community layer are integrated as a one-to-many setting such that many block groups are assigned to one and only one power bus based on their shortest Euclidean distance to the set of power bus candidates. In other words, the population in block groups is clustered such that the locations of power buses are set as the mean of population clusters.

**Figure 2:**
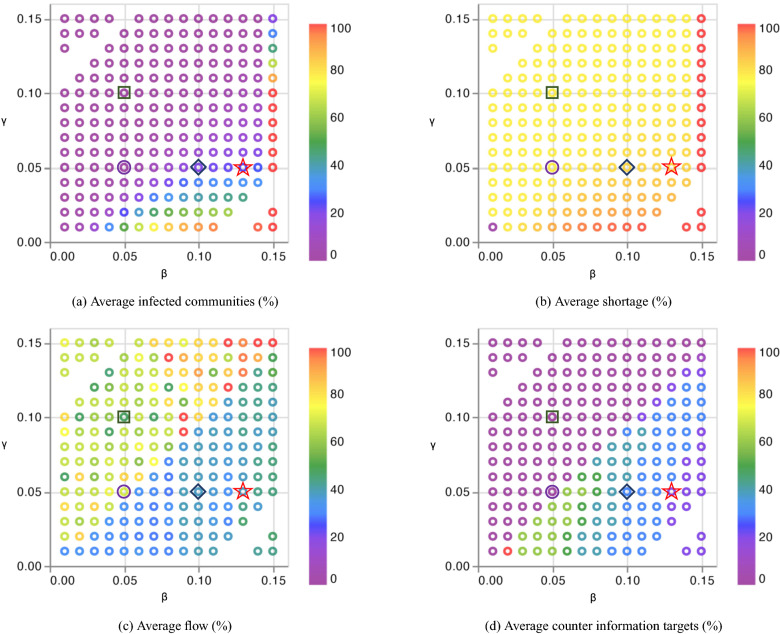
The results of (**a**) average infected communities, (**b**) average shortage, (**c**) average flows, and (**d**) average counter information targets with different combinations of $\beta$ and $\gamma$ as percentage of the baseline value.

We interpret the parameters $\beta$ and $\gamma$ as the rate of disinformation degree of interest and the rate of awareness, respectively. A higher value of $\beta$ results in a higher number of people targeted by disinformation per time period. The higher the value of $\gamma$, the larger the number of people who become aware of disinformation after being targeted per time period. We analyzed the sensitivity of the solutions for different values of $\beta$ and $\gamma$, as shown in Fig. 2. In these graphs, the ideal condition for $\beta$ and $\gamma$ is in the upper left corner of the figures, where the rate of degree of interest takes on the lowest value and the awareness rate is set to its highest value. On the other hand, the worst case is where $\beta$ is relatively higher and $\gamma$ is relatively lower, which is located in the lower right corner of the figures. We ran the model with respect to several different instances of the values $\beta$ and $\gamma$ to analyze the sensitivity of the total shortage, the total number of communities targeted by counter information, the total flow, and the total infected communities. The results are normalized to show the percentage of difference in the resulting values.

To measure the potential spread of disinformation between social media users, the basic reproduction number ($R^0_{i} = \frac{\beta }{\gamma }$) is used. It reveals the expected number of secondary susceptible users that a targeted user can affect with disinformation. For example, given $R^0_{i} = 20$, each newly targeted user is expected to affect 20 secondary users within the community *i*, assuming that all other users contacted are susceptible. To eliminate disinformation or decrease the number of targeted users, the basic reproduction number should satisfy $R^0_{i} < 1$, otherwise disinformation spreads over the network over time ($R^0_{i} > 1$), or the number of targeted users remains constant over time ($R^0_{i} = 1$). These concepts help to understand the situations in which the numerical results are analyzed.

We use the differential equation package^94^ in Julia programming language to solve the SIR model. We used the mathematical optimization modeling language JuMP^95^ to codify our optimization problem in Julia with the optimizer, CPLEX^96^, attached to it. Also, we generated the map in Fig. 1 using QGIS Version 3.24.3^97^.

**Figure 3:**
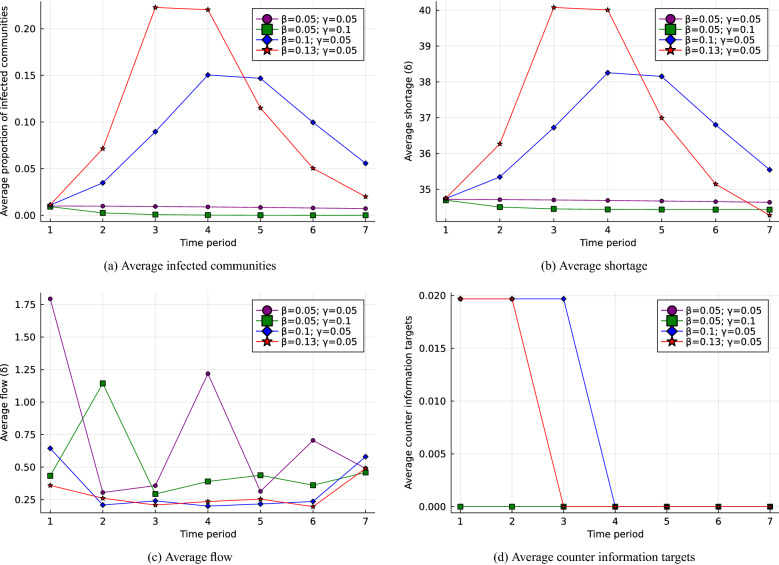
Time series of (**a**) average infected communities, (**b**) average shortage, (**c**) average flow, and (**d**) average counter information targets, with different combinations of $\beta$ and $\gamma$. Note the relationship to particular points in Fig. 2 denoted by shape.

Based on the assumptions discussed previously, we run the model several times to evaluate decisions in different situations of spreading disinformation. The values of the parameters we used to run the model are listed in Table 2, and the results with different combinations of $\beta$ and $\gamma$ are plotted in Fig. 2. As a validation exercise, we optimize the model for different values of disinformation adoption and detection rate based on Raman et al.^80^, who conducted a survey with more than 5000 participants to assess the proportion of people who are expected to adopt and spread disinformation about electricity prices through social networks. They evaluated different scenarios and mapping functions (i.e., linear, quadratic, cubic) to simulate disinformation spread. For simplicity, we adopted the midpoints of the simulated follow-through rates across the mapping functions and performed sensitivity analysis to illustrate the response of the metrics to different scenarios of disinformation propagation in the range. We sampled some notable instances of $\beta$ and $\gamma$ combinations (that is, marked by square, circle, diamond, and star shapes) to analyze the evolution of the corresponding metrics over time plotted in Fig. 3.

**Table 2:** Parameter values.

Parameters	$\rho _{i,t}$	$r^p_{i,t}$	$r_{i,t}$	$n^p_t$	$\bar{t}_t$	Horizon
Values	$-0.7$	0.92	0.2	10	24	169

Figure 2a represents the percentage of infected users in the network with respect to the governing rate of degree of interest in disinformation ($\beta$) and awareness ($\gamma$). Note that there are some empty spots in this figure (and the rest of the figures) as the SIR model is infeasible for some parameter values. The average number of infected users decreases as the awareness rate increases, although the degree of interest rate is sufficiently high in most regions. For the lower awareness rate, there is more potential to have infected communities, and it increases further for higher degree of disinformation of interest.

Figure 2b shows the average network shortage with respect to the governing rate of degree of interest and awareness of disinformation. The average shortage increases as the degree of disinformation interest rate increases for a fixed awareness rate. On the other hand, for a higher awareness rate, the average shortage is lower while the degree of disinformation of interest level is fixed. This trend makes intuitive sense, as we saw in Fig. 2a that the average infected communities decrease with higher levels of awareness, and this means that demand increases less caused by disinformation and, therefore, the average shortage is reduced. The effect of the degree of interest in disinformation is greater than the awareness rate, as it always causes a shortage through the network.

Figure 2c represents the average flow in the network with respect to the governing rate of degree of interest and awareness of disinformation. There is a clear limit in the graph where these two rates are equal ($R^0_{i} = 1$ or $\beta$=$\gamma$). On the lower rectangle of values, where $R^0_{i} > 1$, the basic reproduction number is high enough to allow disinformation to spread over the network in time, and on the upper rectangle of values, where $R^0_{i} < 1$, disinformation has the potential to be eliminated. For the awareness rate above this bound, the average flow is higher in the network, whereas for the region below the bound, we can see much less flow in the network. This is a natural result, as we see that, based on Fig. 2b, the average shortage is lower with greater awareness in the community, and this means that the network can meet demand effectively. In other words, as the average infected communities decreases for a higher awareness rate based on Fig. 2a, the network has more potential to satisfy demands through actual links by transmitting flows.

Figure 2d represents the average number of targeted nodes for counter (good) information with respect to the governing rate of degree of interest and awareness of disinformation. There is a clear limit in the graph where these two rates are equal ($R^0_{i} = 1$ or $\beta$=$\gamma$). For the awareness rate above this bound, the average target counter information is low and not more than 20%. This suggests that if users are at least as aware as the disinformation attracts them, then we can rely less on identifying individuals with whom to supply counter information. This result is in agreement with Fig. 2a and b, as with the higher level of awareness, we have fewer infected communities and also less shortage, which means that less information is needed as a counter mechanism. Furthermore, there are no considerable network shortages that are problematic in this situation. For the lower awareness rate and the higher degree of interest in disinformation, where $R^0_{i} > 1$, the model tries to engage more users with counter information, as shown in Fig. 2d.

We compared the evolution of the metrics discussed over time based on a sample combination of values of $\beta$ and $\gamma$. We sampled two instances for $R^0 > 1$, one for $R^0 < 1$, and another for $R^0 = 1$. We use a normalizing constant, $\delta$, as the units of demands and flows of electric power to interpret the output time series plots. With the time series output plots found in Fig. 3a and b, one would expect similar time series for the average infected communities and the average shortage, because an increase in the number of electric power users results in a more substantial power shortage. Figure 3c shows that despite a relatively higher peak of electric power use for $R^0 > 1$, power demands remain unsatisfied as the average flow remains low relative to scenarios $R^0 < 1$ and $R^0 = 1$. Similarly, the average flow over time remains relatively low for $R^0 > 1$, which means that the network capacity has not been used sufficiently to satisfy the demands in the corresponding scenario of disinformation propagation. This interpretation is also clear from Fig. 3a, since the proportion of communities that adopted disinformation is relatively higher than in two other cases. We also observe that flows fluctuate relatively more in scenarios where $R^0 \le 1$ is average, that is, the use of the network capacity contributed to the satisfaction of demand with a lower spread of disinformation. As Fig. 3d reveals, with a higher intensity of peak demand, we do not necessarily need to target more locations to diffuse counter information. Instead, the duration of disinformation propagation plays a crucial role in selecting the number of communities that become aware of disinformation.

## Concluding remarks

The proposed model aims to analyze the adverse effect of disinformation on electric power networks by integrating (i) an epidemiological SIR model to characterize the spread of disinformation in the communities surrounding electric power nodes and (ii) an electric power network optimization model focusing on minimization of the electric power shortage. In particular, we try to mitigate the effects of disinformation by identifying vulnerable power nodes and countering disinformation spread by targeting particular communities with the spread of (good) information. To illustrate the proposed model, we solved a large-scale electric power network problem associated with Los Angeles County, California.

The evaluation of the results of our proposed model reveals how adversaries can interrupt the performance of critical infrastructures to deliver commodities to customers. In addition, we show how the intensity and duration of disinformation diffusion can be monitored to manage infrastructure performance and make communities counter the disinformation. The proposed model opens up a new space for studying the effect of disinformation diffusion in other infrastructures and managing their performance under a disinformation attack. By applying our proposed model to the large-scale electric power network, under several different scenarios of disinformation diffusion throughout Los Angeles County, we showed how our model can be applied to control the propagation of disinformation projected on the performance of infrastructures.

Due to its criticality, the electric power distribution network is used to illustrate the proposed methodology. However, the proposed integration of epidemiological and network flow models is generally applicable to a wide range of infrastructure networks with appropriate changes to the physical infrastructure flow model (e.g., physical laws that restrict the flow in gas pipelines, user behavior that affects traffic flow in a transportation network).

A primary limitation of this model is the boundary we need to draw to select an electric power distribution network to ensure a timely solution to the optimization problem. However, electric power networks are not isolated, as they interact with each other to mitigate shortages in different stations. With the evolution of computation technology, this model can be applicable and tested on larger-scale networks. Moreover, the proposed model is useful to study the effects of disinformation in other types of critical infrastructure networks, including water and gas, among others, with appropriate physical representations governing the optimization model. Future work includes applying the proposed model to other critical infrastructure networks such as gas distribution systems, nuclear power plants, water distribution systems, etc. To extend the disinformation compartmental model, novel and flexible models (such as agent-based models) can be developed and integrated with the proposed optimization model. Moreover, some parameters used in this article are evaluated by sensitivity analysis or borrowed from previous studies or reports available online. In the future, studies will include a broader range of analysis on the fixed parameters applied to our proposed method. For example, since consumption may not vary linearly in different price ranges during disinformation spread, future work can consider the responsiveness of consumption behavior.

## Data Availability

The datasets generated and/or analyzed during the current study are available in the Github repository https://github.com/jamalzadeh1400/OU_disinformation/tree/cc6e01365e3159c7d7e7b5b1b65ac1706e37b04f.
